# Roles of sulfur-containing compounds in fermented beverages with 2-furfurylthiol as a case example

**DOI:** 10.3389/fnut.2023.1196816

**Published:** 2023-06-30

**Authors:** Guihu Zhang, Peng Xiao, Mengmeng Yuan, Youming Li, Youqiang Xu, Hehe Li, Jinyuan Sun, Baoguo Sun

**Affiliations:** ^1^China Food Flavor and Nutrition Health Innovation Center, Beijing Technology and Business University, Beijing, China; ^2^Key Laboratory of Geriatric Nutrition and Health (Beijing Technology and Business University), Ministry of Education, Beijing, China; ^3^Inner Mongolia Taibus Banner Grassland Brewing Co., Ltd., Xilin Gol League, China; ^4^Beijing Key Laboratory of Quality and Safety, Beijing Technology and Business University, Beijing, China

**Keywords:** 2-furfurylthiol, flavor chemical interaction, Maillard reaction, microorganism, enzyme catalysis

## Abstract

Aroma is a critical component of the flavor and quality of beverages. Among the volatile chemicals responsible for fragrance perception, sulfur compounds are unique odorants due to their extremely low odor threshold. Although trace amounts of sulfur compounds can enhance the flavor profile of beverages, they can lead to off-odors. Sulfur compounds can be formed via Maillard reaction and microbial metabolism, imparting coffee aroma and altering the flavor of beverages. In order to increase the understanding of sulfur compounds in the field of food flavor, 2-furfurylthiol (FFT) was chosen as a representative to discuss the current status of their generation, sensory impact, enrichment, analytical methods, formation mechanisms, aroma deterioration, and aroma regulation. FFT is comprehensively reviewed, and the main beverages of interest are typically baijiu, beer, wine, and coffee. Challenges and recommendations for FFT are also discussed, including analytical methods and mechanisms of formation, interactions between FFT and other compounds, and the development of specific materials to extend the duration of aroma after release.

## 1. Introduction

**Figure 1:**
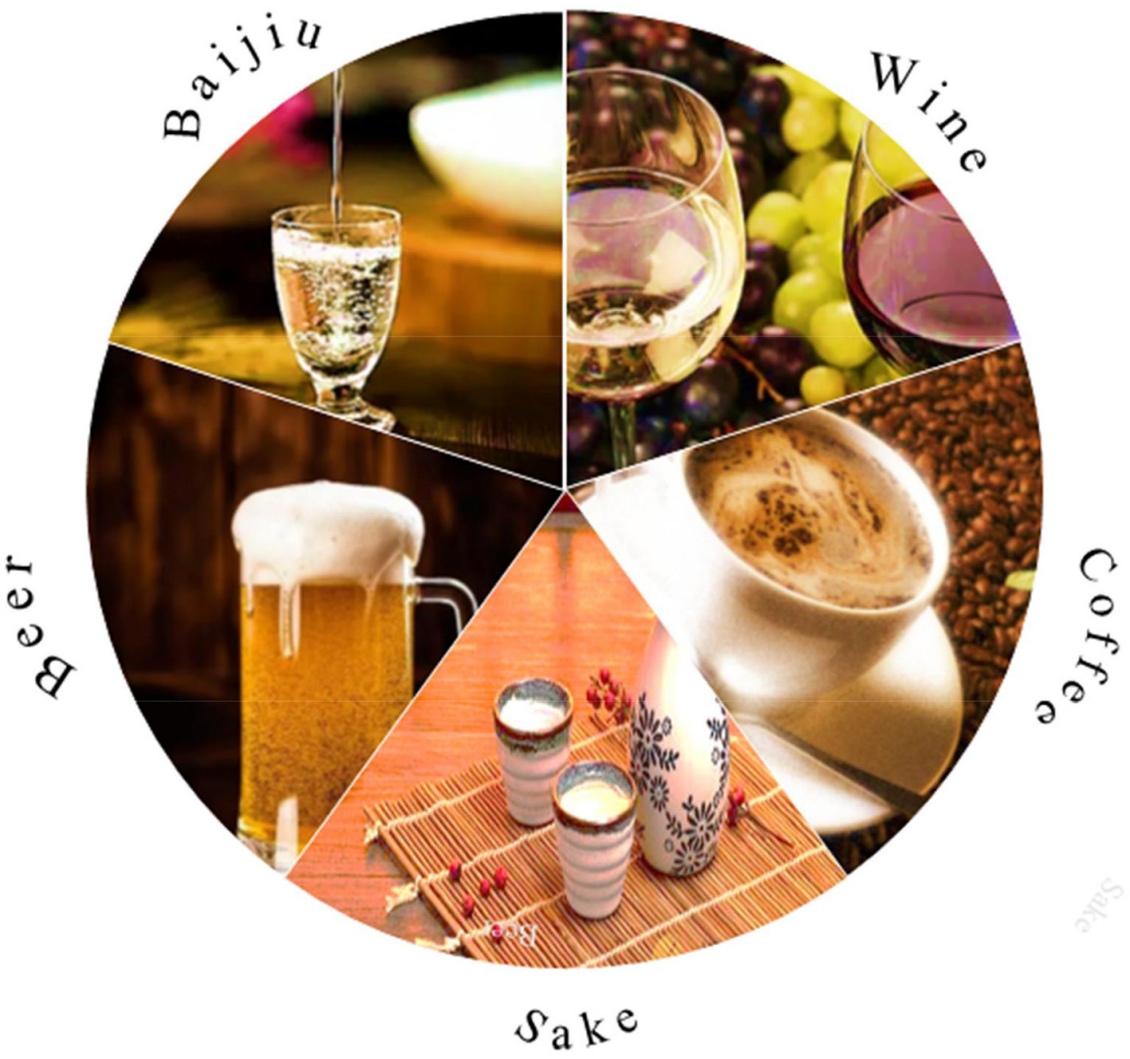
Main liquid matrices enriched with FFT.

Aroma is a crucial factor in the flavor and quality of beverages, and it has a profound impact on the acceptability of consumers. In addition to taste, olfaction plays a vital role in the perception of flavor. During consumption, aroma compounds are released from the foods matrix and transported to the olfactory receptor in the nose, leading to orthonasal and/or retronasal perception, which may affect the experience of consumers (1, 2). A vast number of volatile odorants are released, detected, and discriminated by odorant receptors expressed in the olfactory sensory neurons of the nose (3). This processing is highly accurate, and combined with aroma and other sensor inputs helps to produce complex taste perceptions (4, 5). Previous studies have shown that sulfur-containing compounds can enhance the aroma profiles of beverages with relatively low content (6–11). Among these compounds, FFT is a unique aroma molecule found in many beverages such as coffee (9), grape wine (12), and baijiu (13, 14) (Figure 1), usually formed during fermentation and/or Maillard reaction, and impart aromas of toasted sesame or coffee to the final product.

The significance of FFT lies in its widespread presence in beverages and its potential to influence overall quality including color, flavor, and texture (8, 15–17). Generally, the positive aspects of FFT in beverages can be summarized into the following three points. First, FFT serves as an aroma enhancer at very low thresholds of aroma perception. FFT plays a vital role in the aroma perception of several foodstuffs as a kind of compound with a slight fragrance. For example, fermented soybean paste miso, a traditional Japanese seasoning, actively contributes to the aroma characteristics of miso as a diary element at concentrations ranging from 25 to 409 ng/kg (18). Similarly, baijiu is one of the most popular alcoholic beverages in China. According to previously published data, FFT was found in sauce-flavored, light-flavored, strong-flavored, and sesame-flavored baijiu. Despite its low levels, typically at μg/L, it is also considered to be one of the key aroma compounds that distinguish sesame-flavored baijiu from other odorants substances, as confirmed by aroma recombination and omission analyses (10, 11, 14, 19–21). Moreover, the flavor contribution of FFT to foodstuffs has been demonstrated in other fermented or heat-treated products such as wine (22), sake (23), roasted goose/duck (8, 24), sesame oil (25), Hazelnuts (26), and coffee (9).

Second, FFT can be utilized as a vital compound for classifying different types of characteristic markers in authenticity. Additionally, 2-methyl-3-furanethiol, which has a meaty flavor, and FFT are characteristic aromas of sauce-flavored baijiu and can be used to distinguish sauce-flavored baijiu from light and strong baijiu (14). Similarly, as a characteristic aroma compound in coffee, FFT serves as a quality marker for coffee quality level identification and to distinguish yeast extracts produced at different temperatures, respectively (27, 28). This applies to grading, authenticity, and quality regulation, which are vital in protecting the interests of manufacturers and consumers. Third, perceived interactions between FFT and other compounds in food were identified. Although chlorogenic acid may have a masking effect on the aroma release of FFT in coffee, the interactions between FFT and other molecules are still under-researched (29). The presence of FFT as a flavor factor also influences aroma perception, making it crucial to explore the appropriate content of FFT in beverages.

In addition to boosting the flavor of products, FFT is a double-edged sword, and FFT can enhance the overall flavor of baijiu at low concentrations, but a pickle-like taste is an off-odor in sauce-flavor baijiu that can be degraded by FFT and other volatile sulfur-containing compounds at high concentrations, which are associated with strong odor intensity (30). Nevertheless, to the best of our knowledge, few studies have been conducted on the effect of FFT on the aroma composition of beverages. To better provide researchers with a direction for their research, this study presents the contribution of aroma, discusses the main analytical methods, highlights their advantages and disadvantages, and elucidates the mechanisms of FFT formation in beverages. Our review is expected to increase the understanding of FFT by readers and provide guidance for further research on FFT.

## 2. Occurrence and sensory contribution of 2-furfurylthiol

**Figure 2:**
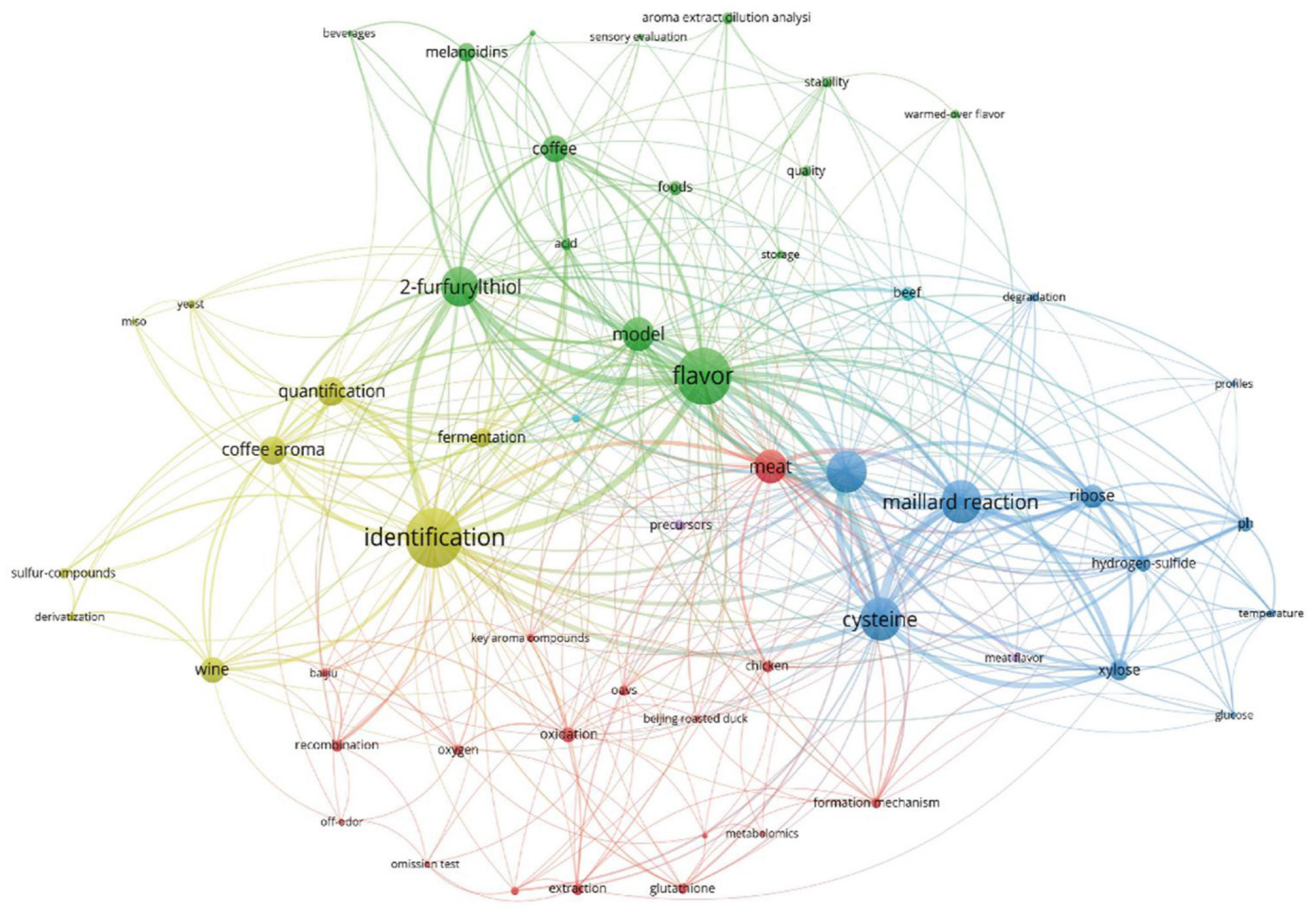
Keyword co-occurrence network diagram in FFT studies in food and beverages, bibliometric diagram of FFT studies visualized in 145 articles retrieved from the Web of Science database published from 2000 to 2022.

A literature review of the Web of Science database showed that 145 articles were published under the keyword “2-furfurylthiol” between 2000 and 2022 (Figure 2). FFT is a sort of compound that contains a sulfhydryl group whose boiling point is always lower than that of the corresponding alcohol and is characterized by a strong odor with a trace odor threshold (31, 32). Typically, FFT is present in diverse fermented or thermally processed beverages (13, 22, 23, 33, 34), as well as in other related foods such as Iberian Ham (35) and squid broth (36). It provides consumers with an enticing and distinctive aroma of roasted coffee or toasted sesame seeds.

FFT, reminiscent of roast meat aroma and roasted coffee aroma or roasted sesame seeds (10, 37), is usually present in baijiu at concentrations below 118 μg/L (38), with a perception threshold equal to 0.1 μg/L in hydroalcoholic solution (10). In addition, FFT always occurs in coffee at a high content level of exceeding 1,000 μg/kg, except for Arabica and Robusta coffee at a concentration close to 0.06~0.18 μg/kg (39–41). However, comprehensively and deeply study of the contribution of aroma compounds to food matrices cannot be achieved by considering the concentration and threshold values alone.

Gas chromatography-olfactometry (GC-O) constitutes the best method for screening odor-active molecules, using the mimic human nose as a detector to identify compounds eluting from the GC column (42). A study of GC-O analysis on baijiu revealed a distinctive odor zone on the column reminiscent of roasted sesame seeds, which was later identified as a contribution from FFT (37). However, olfactometry does not take into account the antagonist and synergic effects between volatiles in a complex matrix such as wine, coffee, as well as aroma enhancers or depressors.

In parallel to olfactometry measurements, the ratio between the concentration of specific volatiles and their perception threshold helps to identify the most active odorants in foodstuffs. Based on a previous study (43), using aroma extract dilution analysis (AEDA), FFT was identified as the main olfactory contributor in roasted white sesame seeds. Thirty common compounds, including FFT, and other 29 chemicals contributed significantly to the aroma profile of coffee brew through the same approach (9). Similarly, a study of Chinese roasted sesame-flavored baijiu revealed the importance of FFT on the flavor of such baijiu (10). To support the qualitative determination, quantitative aspects [e.g., the calculation of odor activity values (OAVs, defined as the ratio of concentration to perception threshold)] have been applied to the identification of influential odors in roasted sesame-flavored baijiu (10, 26).

Finally, odor reconstitution represents the optimal approach to quantitatively measure the contribution of the matrix to the aroma of beverages. A global strategy consisting of qualitative and quantitative determinations of impact odorants followed by omission tests with synthetic aroma models has demonstrated that FFT is the most important odorant in many matrices such as coffee (8, 44, 45). Sensory and quantitative chemical analyses are often carried out in parallel to comprehensively and in-depth characterize odoriferous molecules in foods and beverage matrices.

## 3. Analytical determination of 2-furfurylthiol

The analytical assay of FFT in beverages is mainly hindered by their low concentrations, complexity of the matrices, and susceptibility to oxidative degradation reactions, which result in the rapid conversion of FFT to disulfides through autoxidation or degradation at high-temperature auto-oxidation or degradation at high temperatures (31, 46). In recent years, various methods have been employed to determine FFT in diverse food products, including non-specific techniques applicable to a wide range of volatile compounds, and methods that are selective for FFT. In the following section, common analytical methods for the characterization of FFT in the flavor research area will be discussed, and both the advantages and drawbacks will be highlighted.

### 3.1. Pre-treatment approaches

The concentration, solubility, volatility, and chemical properties of all molecules in food matrices vary widely, resulting in no fixed method for the simultaneous analysis of all substances. Thus, solid phase microextraction (SPME), liquid–liquid extraction (LLE), stir bar sorptive extraction (SBSE), solid phase extraction (SPE), dispersive liquid–liquid microextraction (DLLME), agitator adsorption, supercritical carbon dioxide extraction, and others have been extensively used for the studies in the field of flavor analysis (Table 1). Considering the extremely low content of FFT in matrices, it is difficult to achieve efficient isolation by using conventional extraction/enrichment techniques to analyze FFT due to its instability and possible interactions between FFT and other compounds (54). Therefore, it is of great significance to develop and apply efficient enrichment methods before analysis. SPME is a common, solvent-free technology developed based on SPE and can effectively solve the problems of blocking and channeling existing in SPE and other traditional pre-treatment technologies and requires less samples to handle shorter processing time (47, 59). Generally, SPME can be categorized into headspace solid phase microextraction (HS-SPME) and direct immersion solid phase microextraction (DI-SPME), in which the former is widely used in comparison to DI-SPME (51). HS-SPME has been widely used in various matrices to enrich target odor substances with relatively good results, for instance, HS-SPME has been introduced in wine (12, 48) and coffee (60). Furthermore, it is necessary to select the appropriate extraction fiber due to the discrepancy in extraction efficiency and capacity of the various types of analyte extraction fibers (13). Moreover, it is worth noting that temperature is a non-negligible factor that cannot be ignored due to the tendency of FFT to oxidize or degrade at high temperatures (61, 62).

**Table 1:** Several common pre-treatment methods in flavor analysis.

**Methods**	**Advantages**	**Drawbacks**	**References**
SPME	Solvent-free volatile molecule extraction method, rapid, brief, and low extraction temperature	Extraction of fibers is costly and stability needs to be improved	(47–50)
LLE	Wide range of appropriate substrates, a broad range of extraction solvents, high concentration multiplier, and simple operation	To facilitate the extraction of certain compounds, some of the low boiling point chemicals may be lost during the extraction and concentration process Large amounts of solvent are required and contain environmentally harmful contaminants. Extracted samples will contain small amounts of non-volatile components, contaminating analytical instruments	(51–53)
SPE	High efficiency, wide pH scope, mild operation conditions; enhances sample recovery and handles little volume specimens, effectively separates analytes from interferents, can eliminate matrix effects caused by ethanol, access great reproducibility in quantitative analysis	Complex and costly operation, extraction effect depends on sample matrix and analyte properties, time-consuming	(20, 54, 55)
SBSE	High sensitivity, selectivity, and excellent reproducibility	Poor efficiency, high-budget, sparse and selective coated extraction materials, and stirrers can cause the disproportionation of polar compounds; time-consuming, tedious operation process	(56, 57)
SAFE	Mild extraction conditions and good retention for aromatic substances	Time-consuming and cumbersome to manipulate	(9, 44, 58)
Derivatization reaction	Improved thermal stability of compounds that are difficult to detect and transform for GC detection and also for LC detection; improved extraction recovery, higher detection precision, and lower detection limits	Derivatives make chromatographic separation difficult and can easily introduce impurities or interfering peaks, increasing analytical costs	(31)

Moreover, LLE can extract the analyte and squash the matrix through the analyte solubility divergence between an aqueous sample and water-immiscible organic solvent; ether and dichloromethane are commonly used as extraction solvents. In addition, sensitivity is improved by blowing nitrogen and injecting the final sample for analysis during extraction (51, 63). Pre-concentration FFT of brewed coffee prior to LLE analysis was reported by Casas et.al. Notably, this operation is laborious, tedious, and time-consuming. Especially, the operator will be exposed to hazardous chemicals and, despite the advantage of the small sample size required, some compounds with similar properties may be removed at the same time (64). In addition, the addition of large amounts of organic solvents and inorganic salts to improve the recovery of target compounds may interfere with subsequent chromatographic analysis (52). Fortunately, implantation of liquid–liquid microextraction (LLME) can reduce the volume of solvent required. The choice of solvent depends on the compounds to be extracted and must be safe for the operator and the environment.

Similar to LLE, solvent-assisted flavor evaporation (SAFE) involves the use of organic matter and long extraction times. In contrast to the former, it is a gentle but comprehensive method for extracting volatiles from complex food matrices devised by Engel et al. (58). The SAFE system is a compatible combination of a distillation unit and a high vacuum pump to reduce the loss of heat-sensitive substrates from the sample and to maximize the preservation of the original flavor of the analytes and is particularly suitable for the separation and analysis of volatile molecules in complex matrices (42, 65). SAFE is a highly time-intensive and laborious practice in flavor analysis. The flavor profiles of wine were concentrated before further analysis, despite this method requires significant time and may reduce the loss of target analytes with highly volatile (i.e., FFT) characteristics from the sample (44, 66–68). In addition, unlike SPME, which is commonly used for sample preparation and combined with GC-O analysis, SAFE requires the preparation of a large number of samples (42).

Recently introduced for FFT analysis in wine (56), stir bar sorptive extraction (SBSE) is a novel method for SPME sample pre-treatment. In parallel to SPME, it has a large volume of stationary phase and extraction capacity, allowing for the simultaneous extraction and enrichment of a greater number of trace components without the need for additional stirrers. However, the selection of the type and thickness of the coating on the extraction head is equivalent to the selection of the column in chromatographic analysis (59). Furthermore, in SBSE, only two coatings of polydimethylsiloxane (PDMS) and ethylene glycol silicone (EG silicone) are commercially available (59, 69). In addition, there is a balance between coating thickness and equilibrium extraction time as the adsorption capacity of the analyte is determined. Furthermore, the detection limits are positively correlated with coating thickness and vice versa for the equilibrium extraction time (49, 59). The limited available adsorbents may become a bottleneck for the SBSE technique in FFT analysis, while the availability of new materials (e.g., carbon, metal-organic frameworks, polymers) with high concentration capacity for some specific analytes offers great opportunities for the development of efficient SBSE coatings (51, 70).

In addition to the pre-treatment methods mentioned above, other extraction methods have been used to extract FFT in foodstuffs such as solid phase extraction (SPE) (71, 72), derivatization (64, 73), and simultaneous distillation extraction (SDE) (74). Each method has advantages and disadvantages when used. Therefore, it is necessary to consider the physical–chemical properties of the target compound and the variability of the matrix in order to select the appropriate extraction method to achieve a comprehensive and accurate analysis of FFT in different matrices.

### 3.2. Non-specific technology

Sample preparation is followed by instrumental separation and analysis. With advances in theory and technology, there have been significant improvements in instrumentation for the detection of trace components in the field of flavor analysis. Generally, FFT in various matrices can be separated from the mixture by diverse columns in gas chromatography (GC) or liquid chromatography (LC) and further analyzed by high-sensitivity detectors such as mass spectrometry (MS) and flame ionization detector (FID).

Sulfur compounds are a kind of pivotal trace component in foodstuffs, often produced from sulfur-containing amino acids, and as such, many advanced analytical instruments are used to analyze various sulfur-containing compounds (e.g., FFT). GC-MS technology has become the most widely used technology in flavor component analysis due to its highly commercialized, powerful separation, and qualitative ability (75). Moreover, the qualification and quantification of aroma odorants in GC-MS are based on the divergence of different compounds and the concentration of molecules being related to peak intensity. GC-MS was used to identify FFT from roasted duck and it was regarded as a key aroma molecule (8). However, the detection limit for trace amounts of material is not sufficient, although it is fast, simple, and has fewer solvents. Other instruments are also suitable for the analysis of FFT in complex matrices, such as the gas chromatography pulsed flame photometric detector (GC-PFD), a detector with good selectivity and sensitivity for the analysis of sulfur-containing compounds such as FFT, at low cost. Recently, GC-PFPD was used to qualify and quantify the FFT in baijiu (10). Similar to GC-PFPD, GC-FID and gas chromatography-flame photometric detector (GC-FPD) have been also employed to analyze FFT in diverse matrices. For instance, Zhang et al. (37) applied GC-MS and GC-FPD coupled with GC-O to analyze sesame-flavored volatile compounds in baijiu and detected seven sulfur-containing compounds, including FFT and six other sulfur-containing chemicals (37). In a recent study, volatile thiols, FFT, benzenemethanethiol, and three compounds of ethyl 2-mercaptopropionate were quantified by GC-MS and GC-FPD in four types of heat-treated soy sauce and raw soy sauce (76). Gas chromatography-sulfur chemiluminescence detector (GC-SCD) is the most efficient detector for sulfur-containing compounds with features of high sensitivity, selectivity, and equimolar concentration compared to other detectors such as FPD and PFPD. This stems from the chemiluminescent detection mechanism that sulfur-containing compounds are burned into SO at high temperature and then react with O_3_ to generate an excited state ${\text{SO}}_{2}^{*}$, and when it degrades to the ground state, the excited state SO2^*^ emits a characteristic spectrum (77–79). Due to the complexity of the actual sample composition, samples need to be pretreated for separation before SCD testing. In most applications, researchers combine SCD with GC to improve the purity and sensitivity of the analytes (80). Additionally, gas chromatography-time-of-flight mass spectrometry (GC-TOF/MS) is a versatile detector that is of increasing interest to analysts and industry investors for its fast response, high sensitivity, high accuracy, and high upper limits of mass for the determination of macromolecules (81).

Recently, GC-SCD and GC-TOF/MS were utilized to analyze FFT in wine (67, 74). To overcome the complexity of matrices, and improve peak capacity and separation of aroma compounds, GC × GC systems offer the advantages of increased peak capacity and high resolving power, which are essential for analyzing complex samples (82). For instance, comprehensive two-dimensional gas chromatography/time-of-flight mass spectrometry (GC × GC-TOFMS) and comprehensive two-dimensional gas chromatography-sulfur chemiluminescence detector (GC × GC-SCD) were utilized to characterize the FFT of baijiu and wine (11, 67, 83), respectively. Despite the extremely high sensitivity and response values of SCD detectors for sulfur-containing compounds, their maintenance costs need to be reduced and detection stability needs to be further improved in future studies. Furthermore, their application in practical research less popular since these apparatus (for example, SCD detector) are quite expensive and sensitive to external environmental changes, as well as the high maintenance costs. Additionally, the content of FFT may also experience loss caused by high-temperature GC inlet (generally 250°C) due to the instability when exposed to high temperatures.

### 3.3. Selective methods

The development of analytical approaches and the adaptation of derivatization to achieve more selective, efficient, complex and simplified thiol separation procedures, and/or thiol stabilization have been major developments in thiol separation and resolution. On the one hand, derivatization tempts to hide the sulfhydryl group (or shield a carbonyl group, take 4-mercapto-4-methylpentan-2-one as an example), and the thiol derivatives formed are chemically settled for isolation, as well as thermally stable for GC analysis (84–86). On the other hand, the introduction of substitutions implies that thiol derivatives indicate greater hydrophobicity, lower polarity, or higher proton affinity and may lead to better liquid chromatography (LC) separations and signal enhancement for mass spectrometry (MS)-based detection (54, 85).

In order to retard thiol degradation during analytical procedures, selective derivatization stabilizes the free thiol group. Similarly, for a more selective, efficient, and simplified study of FFTs in flavor analysis, derivatization and/or selective extraction in combination with chromatographic analysis is widely used, as derivatization is more easily extracted, chromatographed, and detected. For this reason, the analysis of FFT frequently implies the use of thiol-specific derivatization agents, such as p-hydroxymercury benzoate (p-HMB) (18) and 4,4′-dithiodipyridine (DTDP) (71).

2,3,4,5,6-pentafluorobenzyl bromide (PFBBr) is a derivative reagent for thiol compounds and is commonly used in the study of thiol compounds. This can be attributed to that the bromine atom is particularly susceptible to nucleophilic substitution by thiols in the presence of a base, thus obtaining PFBBr thiol derivatives desirable for not only stabilizing thiols but also providing electron capture capability and MS detection (87). The derivatization reaction between volatile thiols, such as FFT in wine, and PFBBr has been evaluated in several formats: automated headspace on-fiber derivatization (87), derivatization in organic reagent system (88), and derivatization in SPE column (89). SPME for fiber derivatization is fast, automated, and solvent-free. A polydimethylsiloxane/divinylbenzene (PDMS/DVB) SPME fiber is exposed successively to the vapors of tributylamine (5 min), PFBBr solution (5 min), subsequent, and pre-incubated wine sample (containing ethylenediaminetetraacetic acid, salt, and internal standard) to extraction for 10 min at 55°C (87). This approach provides convenience and less potential interference by using an autosampler and HS-SPME fiber, whereas the linear range of the studied thiols is not very wide and only two thiols (FFT and 3-mercaptohexyl acetate) can be analyzed by this method (87).

Furthermore, several sample preparations may require pH adjustment prior to injection. The p-hydroxymercuribenzoate (p-HMB) selective extraction process is tedious, laborious intensive, and hazardous as it is quite time-consuming due to the need to adjust pH and the use of organic reagents. More importantly, FFT is prone to oxidation during the laborious phase of sample preparation, which may further affect its quantification (23, 90). The FFT of misos were extracted by p-HMB and percolated on a basic anion-exchange column, then washed with high-purity water, released from the FFT-p-HMB complex in the column by using a cysteamine solution (500 mg/50 mL, pH 7), and concentrated and dried in a final step (18). Although this operation can be completed by GC-MS, there is instead room for future modifications of the method due to a large amount of solvent used, time-consuming, and labor-intensive.

Fortuitously, there is a derivatization reagent that can react with FFT in a wide pH range (55, 91). Thiol derivatization with DTDP has also been devised for wine analysis owing to the excellent derivatization ability of sulfhydryl groups at acidic pH, whereby DTDP selectively and rapidly directly reacts with thiols in the natural wine pH range (71). It has been reported that DTDP was used to react with FFT in baijiu (20) and wine (71), followed by SPE coupled with high-performance liquid chromatography-mass spectrometry (HPLC-MS/MS), resulting in excellent result with a limit of detection of 0.7~1.5 ng/L. In coffee, the derivatization of FFT with ebselen has been reported (41). After extraction and derivatization, the concentration of FFT in coffee powder was quantified by applying high-performance liquid chromatography-high resolution mass spectrometry (HPLC-HRMS). Notably, the presence of some important sulfur-containing molecules in coffee powder, such as 4-mercapto-1-butanol and 4-methoxy-2-methyl-2-buthiol, was also determined by the above method (41). Moreover, HRMS provides information on the molecular structure and composition of the molecule, which is crucial in food analysis and contributes to accuracy and reliability (75). The procedure is based on a selective and efficient reaction between thiols and ebselen (selenium-containing reagents), which enables the derivatization and separation of thiols in a time-saving and reduced sample manipulation manner. In summary, a number of key factors need to be considered when selecting the available derivatization reagents, namely reaction specificity and efficiency, matrix complexity and compatibility, sample manipulation required, introduction of interferences, and whether the interferences occur before or after the extraction of the analyte.

Moreover, to mitigate the loss of FFT caused by isolation procedures and decrease quantification errors, solvent extraction and concentration combined with stable isotope dilution assay (SIDA) was utilized (14, 24, 76), another approach is SPME (92, 93). This technique allows for the precise quantification of FFT in samples by using internal standards labeled with stable isotopes, which are often synthesized or purchased using expensive reagents. Recommendations are provided for analytical chemists interested in developing better methods for quantifying FFT in various matrices such as wine, baijiu, and other related matrices. Table 2 provides an overview of general published strategies with sample preparation, types of analysis, and major advantages and drawbacks.

**Table 2:** Analytical methods developed for the analysis of FFT in various matrices.

**Sample preparation**	**Analysis**	**Quantification**	**LOD (μg/L)**	**Advantages (+)/ drawbacks (-)**	**Matrices**	**References**
**Non-specific techniques**						
SPME	GC-MS	External calibration	na	(+) less solvents	Fermentation broth	Zha et al. (94)
HS-SPME	GC-PFPD	Internal calibration ISTD:(4-methylthio-1-butanol)	6.0	(+) less solvents	Baijiu	Sha et al., (10)
HS-SPME	GC × GC-TOFMS	na	na	(+) less solvents	Baijiu	Cheng and Xu (83)
HS-SPME	GC-MS	Internal calibration ISTD:(2-methyl-3 Heptanone)	na	(+) less solvents	Beijing roasted duck	Liu et al. (8)
HS-SPME	GC-MS	ISTD:(2-octanol)	na	(+) less solvents	Fermented fish	Gao et al., (92)
HS-SPME	GC-IMS	ISTD:(2-methyl-3-heptanone)	na	(+) less solvents	Yeast extract	Raza et al., (27)
HS-SPME	GC-MS	Internal calibration ISTD:(1,2-dichlorobenzene)	na	(+) less solvents (+) extremely low limit of quantification	Coffee	Sun et al., (34)
SPME	GC-MS	ISTD:(2-octanol)	na	(+) less solvents	Seasoning product	Li and Liu (15)
LLE	GC × GC-SCD	ISTD:(4-(methylthio)-1-butanol)	0.83 × 10^−3^	(-) organic solvent (+) sensitive to sulfur-containing compounds	Baijiu	Song et al. (14)
LLE	GC-FPD	na	na	(-) organic solvent (-) Time-consuming	Baijiu	Zhang et al. (37)
LLE	GC-MS	Internal calibration ISTD: (4-methoxy-2-methyl-2-mercaptobutane)	na	(-) organic solvents	Soy sauce	Meng et al. (76)
SAFE	GC-MS	Internal calibration ISTD:(1,2-dichlorobenzene)	na	(-) Time-consuming (-) laborious (-) organic solvent	Youtiao	Du et al. (44)
SAFE	GC-MS	Standard addition ISTD:(1-octanol-d18)	na	(-) long extraction time (-) large sample volume (-) hazardous dichloromethane	Roasted goose	Gasior et al. (24)
SAFE	GC-MS	Internal calibration ISTD:(1,2-dichlorobenzene)	na	(-) long extraction time (-) large sample volume (-) hazardous dichloromethane	Pig pork broth	Zhao et al. (68)
SBSE	GC-MS	Internal calibration ISTD: (6MH)	0.36	(-) pH adjustment	Wine	Elpa et al. (56)
**Target methods**						
Extraction with p-HMB and ETP, followed by rinsing on ion exchange resin	GC-MS	Internal calibration ISTD:(ETP derivatives (2FM))	na	(-) Time-consuming (-) organic solvent	Japanese sake	Osafune et al. (23)
Derivatization with DTDP followed by SPE extraction	HPLC-MS/MS	External calibration	0.7~1.5 × 10^−3^	(-) organic solvent (+) Moderate amounts of sample required (+) non-hazardous chemical	Wine	Capone et al. (71)
Extraction with p-HMB, followed by rinsing on SPE and adjusting pH	GC-MS	ISTD:(3-methoxymethylbutanethiol, 3MMB)	na	(-) Time-consuming (-) pH adjustment	Wine	Picard et al. (95)
SPE extraction followed by derivatization with DBU and PFBBr	GC-MS	Internal calibration ISTD:(4-methoxy-R-toluenethiol)	4 × 10^−3^	(-) Time-consuming (-) organic solvent (-) Large sample volume needed	Wine	Mateo-Vivaracho et al. (96)
Derivatization with ebselen followed by LLE extraction	HPLC-MS	Internal calibration ISTD:(4-methoxy-α-toluenethiol)	na	(-) organic solvent (+) extremely low LOQ(0.01ng/L)	Beer and wine	Vichi et al. (97)
Extraction with p-HMB, followed by release from the thiol-p-HMB complex by using cysteamine solution	GC-MS	Internal calibration ISTD:(4-methoxy-2-methyl-2-mercaptobutane)	na	(-) organic solvent (-) pH adjustment (-) Time-consuming (-) laborious	Fermented soybean paste miso	Ohata et al. (18)
HS-SPME	GC-MS	SIDA ISTD:(2-[2H_2_]-furfurylthiol)	na	(+) Simple and fast extraction (+) less solvents (-) expensive reagent	Coffee	Kulapichitr et al. (93)
Derivatization with ebselen followed by extraction with dichloromethane	HPLC-HRMS	Internal calibration ISTD:(4-methoxy-α-toluenethiol)	na	(+) extremely low LOQ (0.1ng/L) (-) hazardous reagent	Coffee	Quintanilla-Casas et al. (64)
Extraction with hexane and derivatization with ebselen	HPLC-HRMS	Internal calibration ISTD:(4-methoxy-α-toluenethiol)	na	(-) organic solvent	Coffee powder	Vichi et al. (41)
SAFE	GC × GC-TOF-MS	SIDA ISTD:(2-[2H_2_]-furfurylthiol)	na	(-) organic solvent (-) Large reagent volume needed (-) time-consuming	Hazelnuts	Kiefl and Schieberle (6)
Derivatization with DTDP and SPE extraction	HPLC-MS/MS	SIDA ISTD: (d_5_-2-furfurylthiol)	na	(-) organic solvent (+) moderate amounts of sample required	Wine	Siebert et al. (12)
Derivatization with DTDP and SPE extraction	UPLC-MS/MS	ISTD:(2-phenylethanethiol) internal calibration	0.001	(+) moderate amounts of sample required (+) low LOD (-) cumbersome operation (-) organic solvent	Baijiu	Yan et al. (20)

na, not available; SIDA, stable isotope dilution assay; DTDP, 4,4-dithiodipyridine; ISTD, internal stand; 6MH, 6-mercaptohexanol; SBSE, stir bar sorptive extraction; DBU, 1,8-diazabicyclo [5.4.0] undec-7-ene; PFBBr, 2,3,4,5,6-pentafluorobenzylbromide; SAFE, solvent-assisted flavor evaporation; Ebselen, 2-phenyl-1,2-benzisoselenazol-3(2H)-one; LOQ, limit of quantification; ETP derivatives (2FM), derivatization of 2FM using ETP; 2FM, 2-furfurylthiol.

## 4. Formation mechanism and manipulation of 2-furfurylthiol

The formation mechanism of FFT is highly complex, as it is influenced by variations in beverage production techniques. Previous studies have identified two key factors for its formation: thermochemical reactions and microbial metabolism (98–100). Hence in this section, relevant studies on the mechanisms of FFT formation in chemical reactions and microbial metabolism are summarized.

### 4.1. Chemical formation mechanism

The formation mechanism of FFT is not fully understood. It is generally accepted that sulfur-containing amino acids are key precursors and sources of sulfur for reactions with sugars and other minor compounds during manufacture (101–103). As early as 20 years ago, a theory was put forward that Maillard-type reactions play an indispensable role in FFT formation (104, 105). In accordance with previous literature, pentoses or hexoses and L-cysteine could produce FFT by producing furfural and H_2_S in a model system (105–107), as demonstrated experimentally by bionic beans (102). In addition, the disturbance factors associated with their production, i.e., the yield of FFT, were also investigated in relation to the factors mentioned above. Temperature is one of the most significant factors in thermal treatment and/or fermentation and dominates manufacturing (108, 109). By adding L-cysteine and ribose to yeast extracts and heating them at high temperatures (100°C and 160°C, respectively), a model of the Maillard reaction was developed, showing that the yield of FFT increased with increasing temperature (27). Model experiments performed by L-cysteine and various carbohydrates at 145°C and 180°C, respectively, confirmed similar results (110). Moreover, pH is an essential factor in deciding the production of FFT. Generally, the amount of FFT is negatively correlated with the change in pH and its production generally increases with decreasing pH. This could also explain, to some extent, the fact that most FFT-rich beverages are acidic matrices (110, 111). Subsequently, the Maillard reaction between L-cysteine and xylose at different pH values from 4.0 to 7.0 confirmed this conclusion and clarified that the yield of FFT increased significantly when the pH was reduced from 7 to 4 (112). Similarly, the yield of FFT, the product of the Maillard reaction, varied depending on the type of carbohydrate. The effect of sugar type on FFT yields has been previously investigated and, in general, the Maillard reaction occurs following high-temperature treatment of L-cysteine and sugar, and the order of concentration of FFT obtained is ribose > xylose > fructose > glucose > rhamnose (107, 110). This phenomenon may be explained by the fact that L-cysteine mainly provides a sulfur source, carbohydrates produce furfural by dehydration, and the structure of pentose seems to have a higher yield/yield of furfural through reaction compared with hexose in terms of structure and composition (113–116). However, it is regrettable that these studies failed to fully investigate the pathways/reactions of furfural and L-cysteine for FFT production at the molecular level. The carbon module labeling (CAMOLA) technical approach is a powerful method to enucleate the formation mechanism of flavor molecules by labeling experiments and isotopomeric quantitation (117).

**Figure 3:**
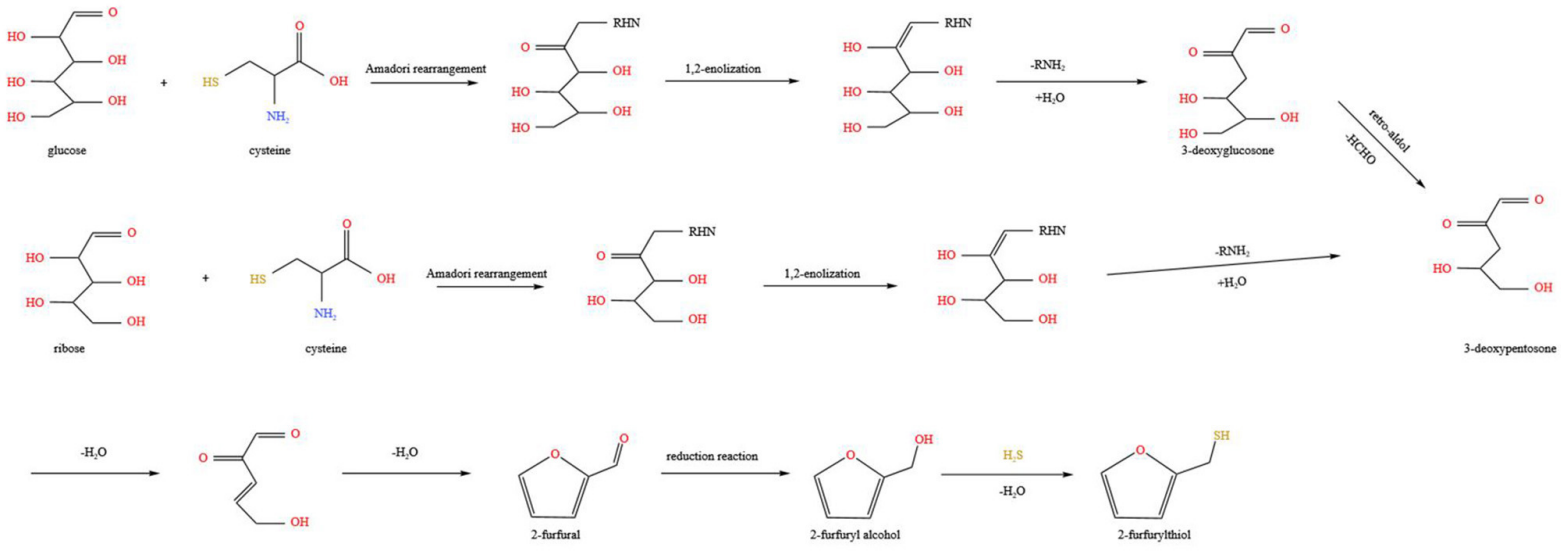
Hypothetical reaction schemes of the formation of FFT from glucose and ribose via Maillard reaction, respectively. Adapted from Liu et al. (99), Zhao et al. (118).

Based on the aforementioned studies, a comprehensive, appropriate, and in-depth study was performed on the model reaction system and real sample system (99). The main pathway for FFT formation involves the Maillard reaction, via dehydration of sugars, as presented in Figure 3 (113, 114). During high-temperature heating, glucose and L-cysteine undergo Amadori rearrangement and 1,2-enolisation to form 3-deoxyglucosone. Further hydroxyl-aldol condensation reactions are carried out, removing one molecule of alcohol and two molecules of water in turn, gradually producing 3-deoxypentosone and the final product furfural. The furfural undergoes a reduction reaction to generate 2-furfuryl alcohol. Finally, H_2_S from the pyrolysis of L-cysteine reacts with 2-furfuryl alcohol to remove one molecule of water, which in turn produces FFT. In addition, previous investigations have suggested that furfural can form FFT by reacting with H_2_S in significantly higher yields than the reaction between L-cysteine and furfural (110). This may be related to the fact that H_2_S is a hydrolysis product of L-cysteine, omitting the hydrolysis reaction step. In addition to thermochemical reactions under thermal treatment conditions, microbial metabolism or enzyme catalysis is essential in the production of FFT. Therefore, the contribution of related microorganisms or enzymes during fermentation will be discussed in the following section.

### 4.2. Microbial metabolism or enzyme catalysis

Microorganisms constitute the complicated micro-ecological environment of the fermentation system, and flavor metabolites generated in the presence of these microflora are closely related to the quality and flavor of fermented beverages (21, 119–121). Yeast, molds, and bacteria are the main microorganisms in the fermentation process. Generally, these members can offer the impetus for the development of fermentation and flavor components. For instance, *Pichia, Saccharomyces, Aspergillus, Rhizomucor*, and *Rhizopus* in jiuqu (a sort of starter in Chinese baijiu), molds in soy sauce, and *Bacillus subtilis* in natto would secrete enzymes to hydrolyze starches and proteins of raw materials (38, 122, 123), respectively, and leads to hydrolysates undergoing Maillard reactions under appropriate conditions. Additionally, microorganisms also consume carbohydrates to produce corresponding metabolites, such as yeast and lactic acid bacteria, which could convert glucose into alcohol and lactic acid (124–126). Consequently, the formation of FFT during fermentation can be a dynamic transversion motivated by the action of microorganisms and enzymes. Conversely, there is still a lack of systematic and comprehensive knowledge regarding the exact role of microbes in the formation of FFT although several studies have been conducted on the formation mechanism of FFT, and some achievements have been obtained.

**Figure 4:**
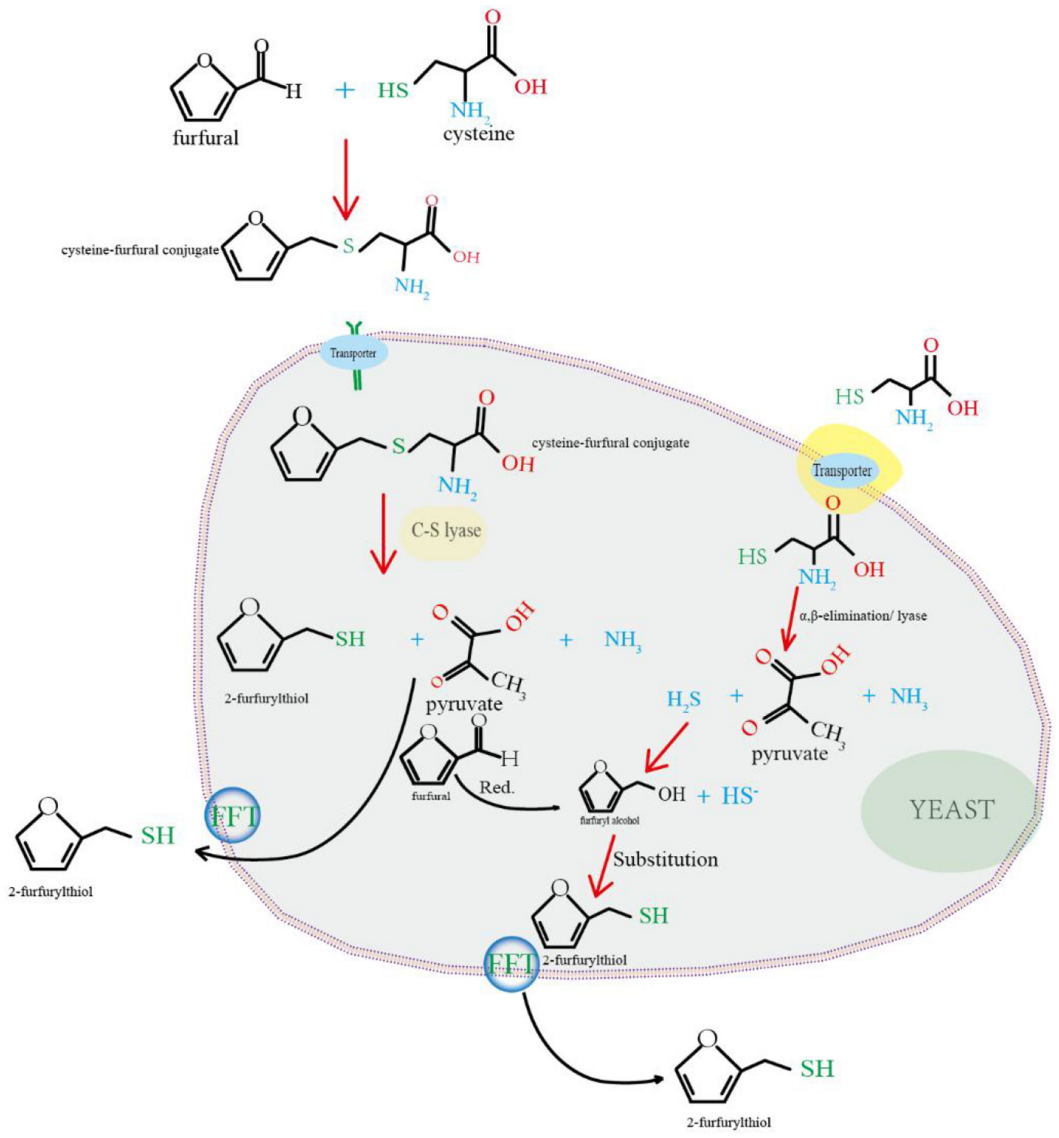
Schematic diagram of the hypothesized biotransformation of FFT.

Based on the preliminary literature research, the biotransformation mechanism of 2-furfurylthiol was summarized and a metabolic production pathway was mapped as follows (Figure 4). In previous reports, furfural and L-cysteine could be used as precursors and produce FFT through yeast metabolism (127). *Pichia, Schizosaccharomyces, Saccharomyces*, and *Zygosaccharomyces* are dominant yeast species in baijiu fermentation, which are essential for the formation of flavor compounds (128). An isolated *S. cerevisiae strain* G20 showed the highest production capacity of FFT with a yield of 3.03 mg/L, and the genes STR3 and CYS3 (encoded cystathionine β-lyase, and cystathionine γ-lyase, respectively) were found to be closely related to FFT synthesis by gene knockout and overexpression verification (94, 129). In addition to flavor compound-producing strain, microbial interactions were found to be an alternative strategy to regulate the formation of flavor compounds during fermentation. *Bacillus subtilis* is an indispensable strain in the fermentation process of baijiu. Amino acids are mainly converted from proteins via the hydrolysis of protease, and *Bacillus subtilis* was one of the major microorganisms producing protease (130). There are synergistic effects between *Bacillus subtilis* and other functional microorganisms. For example, in the fermentation of baijiu inoculated with *Bacillus subtilis* LBM 10019 and *Bacillus vallismortis* LBM 10020, the FFT content in baijiu increased from 1.29 μg/L to 2.44 μg/L after inoculation compared to the non-inoculated group (33). This phenomenon can be explained by the inoculation of *Bacillus*. *Bacillus* inoculation provides a constant supply of L-cysteine and more sulfur sources to the associated FFT-producing strains, thus increasing the FFT content of the final baijiu (131, 132).

In general, there are two main pathways to generate FFT. (1) In the process of alcohol fermentation, mercaptan compounds are released from their non-aromatic precursors through the function of yeast. Although the exact mechanisms by which yeast convert cysteinylated and glutathionylated precursors into the corresponding volatile thiols are still not fully understood, some consensus exists regarding these mechanisms. Previous studies have reported that the uptake of cysteine-3-mercaptohexan-1-ol (3MH) adduct precursors is structurally similar to L-cysteine and is induced by amino acid transport proteins; therefore, it is suggested that furfuryl-cysteine adducts, precursors of FFT, are also induced by amino acid transporters (133, 134). Thus, L-cysteine reacts with furfural to produce furfural-cysteine conjugates (129), which are subsequently transported into the cell by amino acid permeases encoded by genes MUP1, Bap2, Bap3, Gnp1, and OPT1 (encoding Mup1p, branched-chain amino acid permease, amino acid transporter, glutamine permease, oligopeptide transporter, respectively) (134–138). The furfuryl-cysteine adduct is cleaved *in vitro* or after transport into the cell by the C-S lyase encoded by the genes CYS3 and STR3 to produce FFT, pyruvate, and ammonia (129). (2) L-cysteine is transferred into cells under the action of amino acid transporters and then produced as hydrogen sulfide under the guidance of α- and β-elimination reactions or cystathionine β-synthase and cystathionine γ-cleavage enzymes encoded by genes CYS4 and CYS3, respectively (139, 140). Meanwhile, it is worth noting that although L-cysteine is an essential sulfur source of FFT, a high concentration of L-cysteine will restrain the growth of yeast (141). In most cases, the dehydration of carbohydrates produces furfuryl alcohol correspondingly produced by reduction reactions, and the hydroxyl group is replaced by the HS^−^ ion in hydrogen sulfide, ultimately producing FFT, which is secreted extracellularly by the action of carrier proteins (113, 142, 143). It also has been reported that hydrogen sulfide can be used as a precursor to produce FFT from furfural in yeast (100, 142, 144). Many enzymes secreted by microbes in the fermentation environment can catalyze the substrate of materials to generate consequential flavor compounds during growth and metabolism. Lipase from *Candida albicans* served as a biocatalyst to react with S-2-furfuryl thioacetate, and obtained the corresponding product, FFT (145). This finding provides an alternative, environmentally friendly approach to producing natural thiol species and offers theoretical support and technical guidance for generating characteristic flavors in fermented beverages. Future research can focus on the metabolic mechanisms and driving factors of microorganisms and enzymes during catalysis to better regulate the production of FFT during fermentation.

### 4.3. Staling mechanism and regulation of FFT

Aroma plays a crucial role in determining the quality and flavor of a product, as well as influencing individual preferences. However, various external and internal factors lead to the degradation or loss of aroma components in beverages during processing, storage, and transportation. These factors can significantly affect the homogeneity of flavor and quality of the product, ultimately affecting the sensory experience of the consumer and the economic interests of the manufacturer. Correspondingly, we propose to investigate the mechanisms and regulation of aroma deterioration associated with FFT in the following paragraphs.

#### 4.3.1. Staling pathway of FFT

Previous studies have reported that the content of FFT may present unstable/loss problems during production or storage owing to its highly volatile and reactive properties (29, 31, 146). To better understand the degradation mechanism of FFT, the research status on the dissipation mechanism of FFT is discussed.

The fading mechanism of FFT can be categorized into physical and chemical factors. The physical factor involves the irreversible physical diffusion of FFT in coffee, as it is highly volatile and readily evaporates into the headspace in free form, ultimately leading to the loss of sulfur/roast aroma. Nevertheless, chemically induced FFT fading is the result of a combination of reaction mechanisms: ionic and free radical reaction pathways (147).

1. Ionic reaction. Ionic reaction involves electron transfer between the electron donor (nucleophile) and electron acceptor (electrophile) (17). From the perspective of structure, FFT is a strong nucleophilic reagent owing to the existence of sulfhydryl groups of FFT and is easy to participate in the nucleophilic reaction because it is unstable in nature and prone to be oxidized (148). There are several electrophilic addition sites in the coffee matrix, and some of these electrophilic reagents are formed by oxidation. For instance, polyphenols will generate semiquinones and quinones under oxidation conditions (149), while quinones have the potential to add to nucleophiles such as thiols (150, 151). Melanoids, a common macromolecular compound in coffee, contain the 1,4-bis (5-amino-5-carboxy-1-pentyl) pyrazine radical cation (CROSSPY), which can covalently bind to FFT to form conjugates, leading to the degradation of FFT in model systems and in freshly brewed coffee (152, 153). In addition, chlorogenic acid is the main organic acid in coffee, although it does not reduce FFT in studies of model systems (153, 154). Nevertheless, degradation products of chlorogenic hydroxyquinolines can act as aroma-binding precursors for FFT in a semi-simulated reaction system prepared with raw coffee beans, leading to a depletion of FFT (154, 155). Findings similar to the above results were repeated in subsequent studies (156, 157).
2. Free radical reaction. Free radicals can also react with non-volatile components from aroma components or coffee matrices (156). Blank et al. (148) found that the hydroxyl radical generated by hydrogen peroxide and transition metal during brewing coffee could induce FFT to form the corresponding dimer. In addition, the degradation rate of FFT was positively correlated with the activity of hydroxyl radical by investigating the influence of Fenton reaction model conditions on FFT. Subsequently, the model test further proved that furfuryl disulfide is the main oxidation product of free radical reaction (148, 156). Apart from the storage phase, FFT may change significantly during coffee consumption (158). Buettner et al. revealed through model tests that enzymes in saliva can alter the physicochemical properties and sensory characteristics of FFT (158, 159). Initial progress has been made in the study of the rapid decay of FFT caused by non-volatile components in the coffee matrix. In general, it is mainly caused by free radical reaction and ionic reaction (46), and some potential aroma-binding substances have been proposed. Nevertheless, the study of each degradation reaction pathway remains relatively isolated, and the integration and comparative evaluation between each pathway are still insufficient. The main reasons and key components of the rapid degradation of FFT need to be further investigated in future.

#### 4.3.2. Stabilization of FFT

To enhance the stability of holistic aroma during processing and storage, several methods have been proposed to stabilize aroma odor substances. For instance, cyclodextrins are often used as fragrance stabilizers to embed flavor substances to form inclusion complexes, thereby improving the storage stability of volatile flavor substances (160, 161).

Since the staling of FFT in coffee is divided into reversible or irreversible, some studies on the reversible release of FFT have been carried out based on reversible binding studies, and some initial progress has been made on this research topic. Early studies have found that L-cysteine releases FFT bound by the coffee matrix (162), and high concentration of L-cysteine prevents the formation of FFT dimer for quantification in aroma analysis (34). As a hydroxyl radical scavenger, ascorbic acid can be used to mitigate the degradation of FFT caused by free radicals. Furthermore, appropriate anaerobic conditions favor the persistence of aroma compounds. An interesting phenomenon is that FFT exhibits better stability under anaerobic conditions than aerobic conditions (156). Recently, a protective effect provided by ascorbic acid, H_2_S, and wine flavanols has been observed (163). More recently, Sun et al. selected different additives (L-cysteine, ascorbic acid, methionine, sodium sulfite, and glutathione) as aroma-releasing agents; it was finally determined that the addition of 0.045 g/L of L-cysteine and 0.05 g/L of ascorbic acid improved the aroma of fresh coffee and could increase the content of sulfur-containing compounds (17). However, there are relatively few studies investigating the extended aroma duration of FFT. In future, additional studies on aroma modulation by FFT in different matrices could be considered, e.g., the development of food-grade aroma retardants packaged in specialized capsules to prolong the release of beverage aroma.

## 5. Perspectives and conclusion

Aroma is a key factor in the flavor and quality of food products, and the perception of aroma plays an important role during consumption. Challenges in FFT investigation are the need to modify analytical methods, including specific extraction and detection due to trace concentrations, non-uniform distribution in the same/similar species, and interactions between FFT and other species in the matrix. Extraction processes often need to be fast, solvent less, efficient, and simple, while detection often requires low detection limits and high stability due to low FFT concentrations. In terms of specific extraction, currently, available approaches can be coupled with the popular MS detection for sensitive analysis of FFT. Appropriate analytical methods can provide more accurate and comprehensive information, as this is a primary prerequisite for flavor studies. FFT has a pivotal effect on the flavor and quality of beverages. Beverages have long been consumed as an integral part of the daily human diet. Nevertheless, much less attention has been paid to FFT in fermentation matrices than in thermal treatments and model systems. This review provides the first systematic review of FFT. The presence of FFT is the result of diverse factors that affect the precursor concentrations. This occurs at all levels: (i) in raw materials, (ii) released during heat treatment and/or fermentation, and (iii) stored under convenient conditions until the final product is consumed.

The presence of precursors in crude materials depends on several factors, such as the type and content of amino acid and sugar, vine management, and maturity. These aspects are currently well-known, even if the mechanisms implicated are not fully elucidated. The conversion of these precursors into FFT during heating and/or fermentation remains the key step and the subject of most research in this field. Studies carried out by research groups worldwide have been able to identify many precursors and determine their conversion mechanisms, depending on the composition of the substance (i.e., the type of sugars and amino acids and the location of the precursors in the matrix), as well as yeast genetic information and process conditions. In most of these studies, organic chemistry is necessary for the identification and quantification of FFT and its precursors and for the better comprehension of yeast contributions and physiology. As the discovery of some precursors is new, many aspects in this field have not been comprehensively investigated. After final products are obtained, all of the technology employed must focus on preventing oxidation and binding upon the release of the FFT. Consequently, chemistry and biochemistry offer the only tool to understand the various mechanisms (oxidation or nucleophilic substitution) and therefore propose ways to avoid the loss of aroma due to the disappearance of these compounds. Our review helps the academic community to grasp the current state of research and informs further studies on FFT in beverages.

## Author contributions

GZ investigation, methodology, writing—original draft, and writing—review and editing. PX, MY, and BS: writing—review and editing. YX: data curation, formal analysis, writing—original draft, and writing—review and editing. HL: supervision, validation, and writing—review and editing. YL: project administration and resources. JS: supervision. All authors contributed to the article and approved the submitted version.
